# Valve-specific structural vulnerability in infective endocarditis: a narrative review of surgical complexity and outcomes

**DOI:** 10.1186/s13019-026-04523-w

**Published:** 2026-07-17

**Authors:** Najmi Hakim Abd Rani, Afifah Mohamed, Zaleha Md Isa, Suhaini Kadiman, Taty Anna Kamarudin

**Affiliations:** 1 Non-Invasive Cardiovascular Laboratory, National Heart Institute, Kuala Lumpur, Malaysia; 2 Centre for Diagnostic, Therapeutic & Investigative Studies (CODTIS), Faculty of Health Sciences, Universiti Kebangsaan Malaysia (UKM), Kuala Lumpur, Malaysia; 3 Department of Public Health Medicine, Faculty of Medicine, Universiti Kebangsaan Malaysia (UKM), Kuala Lumpur, Malaysia; 4 Department of Anaesthesiology and Critical Care, National Heart Institute, Kuala Lumpur, Malaysia; 5 Department of Anatomy, Faculty of Medicine, Universiti Kebangsaan Malaysia (UKM), Kuala Lumpur, Malaysia

**Keywords:** Infective endocarditis, Cardiac valve anatomy, Periannular abscess, Valve repair, Prosthetic valve endocarditis, Surgical outcomes, Cardiac surgery, Structural vulnerability, Peri-annular extension

## Abstract

**Background:**

Infective endocarditis (IE) carries in-hospital mortality of 15–20% and 1-year mortality approaching 40%, with surgery required in 40–50% of cases. IE is not uniformly distributed across cardiac valves aortic 30–45%, mitral 25–30%, tricuspid 5–10%, pulmonary < 2%, PVE 10–30% and this distribution reflects valve-specific anatomical determinants governing surgical complexity and outcomes. Despite the 2023 ESC Guidelines, a unified structural framework linking anatomical vulnerability to surgical decision-making remains absent.

**Objective:**

To propose a valve-specific structural vulnerability framework and examine its implications for surgical complexity, perioperative outcomes, and re-surgery risk across all cardiac valve types.

**Methods:**

A narrative review of peer-reviewed literature (2015–2025) was conducted across PubMed, Scopus, and MEDLINE. Systematic reviews, meta-analyses, multicentre registries, and guideline documents were synthesised narratively.

**Key Findings:**

A structural vulnerability gradient (prosthetic valves > aortic > mitral > tricuspid > pulmonary) is proposed as a conceptual model of valve-specific disease behaviour. Aortic IE carries maximal vulnerability through peri-annular extension, 30–40% of cases, requiring radical root reconstruction with elevated operative mortality. Mitral IE exhibits conditional vulnerability; repair yields superior outcomes over replacement. Tricuspid IE is predominantly IVDU-associated with comparatively lower operative mortality. PVE carries the highest operative risk, with prolonged operative times and significant reoperation rates.

**Conclusions:**

IE is best understood as a valve- and substrate-specific disease in which anatomical determinants drive surgical complexity and clinical prognosis. This proposed vulnerability hierarchy may enable preoperative risk stratification, inform valve-specific surgical planning, and provide a mechanistic framework for understanding differential outcomes across valve types. Prospective outcome registries and standardised complexity metrics are needed to validate and refine this conceptual model.

**Supplementary Information:**

The online version contains supplementary material available at https://doi.org/10.1186/s13019-026-04523-w.

## Background

Infective endocarditis (IE) remains a life-threatening cardiovascular infection with in-hospital mortality of 15–20% and 1-year mortality approaching 40% despite advances in antimicrobial therapy, diagnostic imaging, and surgical techniques [1–3]. The disease is characterised by microbial colonisation of cardiac valve tissue, formation of vegetations composed of platelets, fibrin, and microorganisms, and progressive valvular destruction that frequently necessitates surgical intervention in 40–50% of cases [1, 4, 5]. The 2015 European Society of Cardiology (ESC) Guidelines for the management of IE [1] and the updated 2023 ESC Guidelines [2] provide comprehensive algorithms for diagnosis, antimicrobial therapy, and surgical timing, establishing a robust framework for clinical decision-making.

However, a fundamental epidemiological observation challenges the notion of IE as a uniform disease process: the distribution of IE across cardiac valves is distinctly non-random. Large prospective cohort studies consistently demonstrate that the aortic valve accounts for 30–45% of native valve IE, the mitral valve 25–30%, the tricuspid valve 5–10%, and the pulmonary valve less than 2%, with prosthetic valve endocarditis (PVE) comprising 10–30% of all IE cases [1, 3, 10]. This striking disparity in valve involvement cannot be attributed solely to differences in microbial virulence or host immune status; rather, it suggests intrinsic valve-specific anatomical and hemodynamic determinants that fundamentally influence susceptibility to infection, patterns of tissue destruction, and subsequent clinical outcomes.

While current guidelines comprehensively address management strategies—including indications for surgery, antimicrobial regimens, and timing of intervention—they do not systematically integrate the anatomical substrate underlying valve-specific vulnerability. The guidelines appropriately focus on clinical decision-making algorithms, but the mechanistic understanding of why certain valves are preferentially affected, why specific patterns of destruction occur, and how anatomical factors dictate surgical complexity remains incompletely synthesised in the literature. This knowledge gap has important implications: surgical planning, risk stratification, and prognostic counselling all depend on understanding not only the presence of IE but also the specific valve involved and the extent of structural destruction.

This review introduces and applies the concept of a structural vulnerability gradient as its central organising framework, prosthetic valves > aortic > mitral > tricuspid > pulmonary (native). This gradient, derived inductively from the synthesis of anatomical, hemodynamic, and surgical outcome data, is proposed as a conceptual model to help explain the non-random distribution of IE across cardiac valves and to inform reasoning about surgical complexity, procedural risk, and re-surgery probability. The gradient is referenced throughout this manuscript as a mechanistic lens for interpreting valve-specific evidence.

The aim of this narrative review is to reinterpret IE through a valve-specific anatomical lens, proposing a structural vulnerability gradient as a conceptual framework to help explain the differential susceptibility, patterns of destruction, and surgical outcomes across cardiac valves. We systematically examine how anatomical determinants, including hemodynamic stress, fibrous cardiac skeleton architecture, tissue composition, and spatial relationships, influence surgical technique selection, procedural complexity, clinical outcomes, and re-surgery risk. By integrating anatomical foundations with contemporary surgical data, this review provides a mechanistic framework for understanding valve-specific disease behavior and offers practical insights for preoperative planning and risk stratification.

## Review methodology

### Search strategy and databases

This narrative review was conducted in accordance with the Scale for the Assessment of Narrative Review Articles (SANRA) framework, which provides methodological rigour for non-systematic reviews [16]. A structured literature search was performed across three major biomedical databases, PubMed/MEDLINE, Scopus, and the Cochrane Library, covering publications from January 2000 to December 2024, with particular emphasis on studies published between 2015 and 2024 to reflect contemporary surgical practice and updated guideline recommendations. The search was conducted in October–November 2024.

The primary search terms were constructed using Medical Subject Headings (MeSH) and free-text terms in the following combinations: (“infective endocarditis” OR “bacterial endocarditis”) AND (“aortic valve” OR “mitral valve” OR “tricuspid valve” OR “pulmonary valve” OR “prosthetic valve”) AND (“surgical treatment” OR “valve surgery” OR “valve repair” OR “valve replacement” OR “homograft” OR “root reconstruction”). Secondary searches were performed for specific topics: (“peri-annular abscess” OR “intervalvular fibrous body”), (“TAVI endocarditis” OR “transcatheter valve endocarditis”), (“structural vulnerability” OR “anatomical determinants”), (“homograft versus prosthesis” OR “cryopreserved homograft”), (“repair versus replacement” OR “mitral repair endocarditis”), and (“imaging endocarditis” OR “cardiac CT endocarditis” OR “PET endocarditis”). Reference lists of identified systematic reviews and meta-analyses were hand-searched to identify additional relevant studies.

### Inclusion and exclusion criteria

(1) Original research articles, systematic reviews, and meta-analyses reporting surgical outcomes in IE stratified by valve type; (2) studies with ≥ 50 patients undergoing surgery for IE; (3) studies reporting at least one primary outcome (30-day/in-hospital mortality, freedom from reoperation, or 5-year survival); (4) international clinical guidelines from the European Society of Cardiology (ESC 2015, 2023) and the American Heart Association (AHA); (5) imaging studies evaluating the diagnostic performance of TEE, cardiac CT, and nuclear imaging in IE; (6) studies published in English.

(1) Case reports and case series with fewer than 20 patients; (2) studies focused exclusively on medical management without surgical data; (3) paediatric IE studies (age < 18 years); (4) studies reporting only microbiological outcomes without clinical or surgical endpoints; (5) non-English language publications without available translation; (6) conference abstracts without full-text publication.

### Data synthesis and quality assessment

Given the heterogeneous nature of available evidence, predominantly retrospective single-center cohort studies, with a limited number of multicenter registries and meta-analyses a formal meta-analytic synthesis was not performed. Instead, a narrative synthesis approach was adopted, prioritising data from multicentre registries (≥ 500 patients), systematic reviews with meta-analyses, and guideline-endorsed recommendations. Where multiple studies reported the same outcome, pooled estimates from meta-analyses were preferentially cited. For outcomes without meta-analytic data, ranges from the two largest available cohort studies are reported.

The structural vulnerability gradient, the proposed organising framework of this review was developed inductively from the synthesis of anatomical, hemodynamic, and surgical outcome data across valve types. Vulnerability was operationalised as a composite construct incorporating: (1) prevalence of IE at each valve relative to anatomical exposure, (2) rate of peri-annular extension and complex destruction, (3) operative complexity (CPB time, cross-clamp time, reconstruction technique), and (4) perioperative mortality. This conceptual model is intended to organise clinical evidence and guide surgical reasoning rather than to serve as a validated prognostic score.

## Anatomical foundations of valve vulnerability

### Hemodynamic stress and endothelial injury

The fundamental prerequisite for IE is endothelial injury, which exposes subendothelial collagen and initiates platelet-fibrin deposition to form non-bacterial thrombotic endocarditis (NBTE) [15, 16]. Hemodynamic stress is the primary driver of endothelial injury, with high-velocity turbulent flow generating shear forces that disrupt the endothelial monolayer. The magnitude of hemodynamic stress varies dramatically across cardiac valves: left-sided valves (aortic and mitral) experience systolic pressures of 120 mmHg and diastolic pressures of 80 mmHg, whereas right-sided valves (tricuspid and pulmonary) operate in a low-pressure environment with systolic pressures of 25 mmHg and diastolic pressures near zero [15, 33].

Rodbard’s principle, established through experimental models, demonstrates that bacterial colonisation occurs preferentially on the low-pressure side of regurgitant jets, where high-velocity flow impacts the endothelium [16]. In aortic regurgitation, vegetations form on the ventricular surface of the aortic valve leaflets; in mitral regurgitation, vegetations develop on the atrial surface of the mitral leaflets. This spatial pattern reflects the site of maximal endothelial injury and subsequent NBTE formation. The sustained high-pressure environment of left-sided valves not only increases baseline endothelial stress but also amplifies the impact of pre-existing structural abnormalities such as calcific degeneration, myxomatous degeneration, or congenital malformations that further disrupt laminar flow and create turbulent eddies conducive to bacterial adherence [15, 18].

### Structural substrate and tissue composition

The fibrous cardiac skeleton forms the structural foundation of the heart, comprising four fibrous rings (annuli) that anchor the valve leaflets and provide electrical insulation between atria and ventricles [14, 17]. The central fibrous body, located at the confluence of the aortic, mitral, and tricuspid annuli, and the right and left fibrous trigones represent dense collagenous structures that are relatively avascular and immunologically privileged [14]. This anatomical arrangement has profound implications for IE, infection involving the fibrous skeleton is difficult to eradicate with antimicrobial therapy alone due to limited vascular penetration, and surgical debridement often requires extensive reconstruction [6, 7, 17].

Valve leaflets themselves are composed of three histological layers: the fibrosa (dense collagen providing structural integrity), the spongiosa (loose connective tissue with glycosaminoglycans allowing flexibility), and the ventricularis or atrialis (elastin-rich layer enabling elastic recoil) [22]. Critically, valve leaflets are avascular structures that rely on diffusion from surrounding blood and synovial-like fluid for nutrient supply [22]. This avascularity renders valve tissue inherently vulnerable to infection: once bacteria establish a foothold within the leaflet substance, antimicrobial penetration is limited, and the host immune response is attenuated. The relative proportions of collagen and elastin differ across valves, with the aortic valve containing a higher collagen-to-elastin ratio to withstand systolic pressure, whereas the mitral valve has greater elastin content to accommodate the complex deformation during ventricular contraction [22, 23].

### Spatial relationships and extension patterns

The spatial relationships between cardiac valves and adjacent structures determine the patterns of infection extension and the complexity of surgical reconstruction. The aortic valve is intimately related to the mitral valve through the aortomitral continuity, a fibrous structure connecting the non-coronary cusp of the aortic valve to the anterior leaflet of the mitral valve [14, 17]. This anatomical continuity explains the propensity for aortic IE to extend into the intervalvular fibrous body (IFB) and subsequently involve the mitral valve, creating complex double-valve endocarditis that requires extensive reconstruction [6, 7, 70].

The right and left fibrous trigones, located at the commissures between the non-coronary and left coronary cusps (right trigone) and between the non-coronary and right coronary cusps (left trigone), serve as anatomical highways for infection spread [17]. Peri-annular abscesses originating in the aortic root can track along these fibrous structures to involve the conduction system (atrioventricular node and bundle of His), resulting in heart block, or extend into the atria and ventricles to form aorto-cavitary fistulae [6, 7]. The tricuspid valve, in contrast, is relatively isolated from the fibrous skeleton and lacks direct continuity with other valves, which may partially explain its lower propensity for complex extension patterns despite its anatomical proximity to the conduction system [13, 14].

## Aortic valve: maximal vulnerability and surgical complexity

### Anatomical determinants of aortic valve susceptibility

The aortic valve demonstrates the highest intrinsic vulnerability to IE among native cardiac valves, accounting for 30–45% of all native valve IE cases [1, 3]. This susceptibility reflects the convergence of high hemodynamic stress, age-related calcific degeneration (nearly universal after age 65), and bicuspid aortic valve (BAV) morphology present in 1–2% of the population and conferring a five-fold increased IE risk [15, 18]. Critically, fibrous skeleton continuity, particularly the aortomitral continuity and central fibrous body, provides a direct anatomical conduit for infection to extend beyond the leaflets into peri-annular structures [6, 7, 17].

### Patterns of destruction and peri-annular extension

Aortic valve IE is characterised by aggressive tissue destruction and a high propensity for peri-annular extension. Peri-annular abscess, defined as a suppurative cavity within the fibrous skeleton or adjacent myocardium, occurs in 30–40% of aortic IE cases and represents a major determinant of surgical complexity and mortality [6, 7]. Abscesses typically originate at the base of the aortic cusps, where the leaflet insertion meets the fibrous annulus, and extend along the fibrous skeleton into the intervalvular fibrous body (IFB), the mitral-aortic continuity, or the ventricular septum [6, 7, 17].

Aorto-cavitary fistulae develop in 1.6–3.2% of aortic IE cases, arising from progressive abscess rupture into adjacent chambers and causing severe hemodynamic instability [6]. New-onset atrioventricular (AV) block is pathognomonic of conduction system involvement via the membranous septum adjacent to the non-coronary cusp, and mandates urgent surgical evaluation [6, 7].

### Surgical technique and procedural complexity

Radical debridement of all infected and necrotic tissue is the cornerstone of surgical management for aortic IE, with the extent of destruction dictating the complexity of reconstruction [12, 19–21, 71, 72]. For localised disease confined to the valve leaflets without peri-annular extension, aortic valve replacement (AVR) with a bioprosthetic or mechanical valve is the standard approach. However, the presence of peri-annular abscess, fistula formation, or extensive annular destruction necessitates more complex reconstructive techniques.

For extensive root destruction, the Bentall procedure (composite valved conduit with coronary reimplantation) or cryopreserved aortic homograft root replacement is employed [19–21, 71, 72]. Homografts are preferred by many centres for active IE, as they replace infected tissue with viable allograft, resist reinfection, and avoid prosthetic material in a contaminated field.

The commando procedure is reserved for the most complex cases involving destruction of the IFB with extension to the mitral valve [70, 72]. This operation requires radical debridement of the IFB, reconstruction with pericardial or homograft patches, and often concomitant mitral valve repair or replacement. The technical demands of these procedures are reflected in prolonged operative times: cardiopulmonary bypass (CPB) times for homograft root replacement are reported across published series in the range of approximately 190–250 min, with cross-clamp times of approximately 140–180 min [71]. For aorto-cavitary fistula repair, CPB times are substantially prolonged (reported mean exceeding 230 min in single-centre series), reflecting the complexity of identifying and closing the fistulous tract while reconstructing the aortic root [72].

### Clinical outcomes and prognostic factors

The clinical outcomes of aortic IE surgery are heavily influenced by the extent of peri-annular destruction and the complexity of reconstruction required. For isolated aortic valve replacement without peri-annular extension, 30-day mortality ranges from 8 to 12% [12, 75]. However, the presence of peri-annular abscess increases 30-day mortality to 18–22% for homograft root replacement [71, 75]. Intervalvular fibrous body reconstruction carries 30-day mortality of 28.3% and 90-day mortality of 37.0%, reflecting the extreme complexity and hemodynamic instability associated with these cases [72].

Long-term survival after aortic root replacement for IE is approximately 58–65% at 5 years, with freedom from reoperation of 85–90% at 10 years [71, 74, 86]. Independent predictors of mortality include S. aureus infection (OR 3.2–4.8), left ventricular EF < 35%, peri-annular abscess (OR 2.1), and emergency surgery (OR 2.8) [12, 71, 75]. Prosthesis choice, homograft versus bioprosthetic versus mechanical remains debated; some series report lower reinfection rates with homografts but no consistent survival advantage [73–76].

## Mitral valve: conditional vulnerability and repair potential

### Anatomical basis of mitral valve involvement

The mitral valve accounts for 25–30% of native valve IE cases, representing the second most commonly affected valve [1, 3]. Unlike the aortic valve, which demonstrates intrinsic vulnerability due to high hemodynamic stress, the mitral valve exhibits conditional vulnerability: IE typically occurs in the setting of pre-existing structural abnormalities such as mitral valve prolapse (MVP), rheumatic heart disease, or degenerative mitral regurgitation [22–26]. MVP, present in 2–3% of the population, is the most common predisposing condition in developed countries, conferring an eight-fold increased risk of IE compared to the general population [25].

The anatomical basis of mitral valve vulnerability relates to Rodbard’s principle: regurgitant jets create high-velocity turbulent flow that impacts the atrial surface of the mitral leaflets, causing endothelial injury and NBTE formation [16, 23]. The mitral valve apparatus is structurally complex, comprising the anterior and posterior leaflets, the mitral annulus, the chordae tendineae, and the papillary muscles [22]. This complexity provides multiple potential sites for infection: leaflet tissue, chordal attachments, and the annulus. The anterior leaflet, which is in fibrous continuity with the aortic valve through the aortomitral continuity, is particularly vulnerable to extension from aortic IE [6, 7, 22].

### Patterns of destruction and repair feasibility

Mitral valve IE demonstrates diverse patterns of destruction that critically influence repair feasibility. Leaflet perforation, the most common lesion, results from direct bacterial invasion and enzymatic degradation of leaflet tissue, creating regurgitant orifices that range from small perforations to large defects [27, 30, 78]. Chordal rupture, caused by infection of the chordae tendineae or secondary to leaflet destruction, results in flail leaflet segments and severe mitral regurgitation [22, 30]. Annular abscess, occurring in 10–15% of mitral IE cases, involves the fibrous annulus and may extend into the aortomitral continuity or the left atrial wall [7, 22].

The feasibility of mitral valve repair depends on the extent and location of destruction. Isolated leaflet perforations, particularly in the posterior leaflet, are highly amenable to repair using pericardial patch techniques [80]. Chordal rupture can be addressed with chordal transfer, artificial chordae implantation, or leaflet resection with annuloplasty [30, 78, 79]. However, extensive destruction involving multiple leaflet segments, large annular abscesses, or severe calcification often precludes repair and necessitates valve replacement [8, 30].

The anterior leaflet, due to its larger surface area and fibrous continuity with the aortic valve, is more challenging to repair than the posterior leaflet [22, 78]. Extension from aortic IE into the mitral valve via the aortomitral continuity typically involves the anterior leaflet and often requires double-valve surgery with complex reconstruction [6, 7, 70].

### Surgical technique: repair versus replacement

The fundamental surgical decision in mitral IE is whether to repair or replace the valve. Mitral valve repair is strongly preferred when feasible, as it preserves the native valve apparatus, maintains left ventricular geometry through preservation of the subvalvular apparatus, and avoids the need for long-term anticoagulation, if a bioprosthesis would otherwise be used [8, 30, 77–79]. Repair techniques include leaflet debridement with pericardial patch reconstruction, chordal replacement or transfer, leaflet resection (particularly for posterior leaflet), and annuloplasty with a prosthetic ring to restore leaflet coaptation [78–80].

Mitral valve replacement is indicated when destruction is too extensive for repair, when repair attempts fail intraoperatively, or when there is significant annular involvement precluding stable repair [8, 30]. In the setting of active infection, bioprosthetic valves are generally preferred over mechanical valves due to lower risk of prosthetic valve endocarditis (PVE) recurrence, although this remains debated [29, 30]. The choice between repair and replacement has profound prognostic implications.

A landmark systematic review and meta-analysis by Feringa et al. [8] analysed 1,076 patients undergoing surgery for mitral IE and demonstrated pooled early mortality of 3.7% for repair versus 10.9% for replacement. Long-term survival was significantly better with repair, with an adjusted hazard ratio (HR) of 0.70 (95% confidence interval [CI] 0.55–0.89) favouring repair [8]. A systematic review of tricuspid valve IE surgery by Yanagawa et al. [81] similarly reported that valve repair was associated with lower recurrent IE (RR 0.17) and reduced need for reoperation (RR 0.26) compared to replacement, supporting the preference for repair across valve types where technically feasible. These data establish mitral valve repair as the gold standard when technically feasible.

### Clinical outcomes and prognostic factors

Operative mortality for mitral IE surgery ranges from 5 to 15%, depending on the complexity of the procedure and patient comorbidities [8, 30, 77, 81]. Factors associated with increased mortality include S. aureus infection (OR 3.5 compared to streptococcal IE), left ventricular EF less than 35%, concomitant aortic valve surgery (double-valve endocarditis), and emergency surgery [8, 30, 77]. The presence of annular abscess increases operative risk but does not preclude repair if the abscess can be adequately debrided and reconstructed [7, 30].

Long-term outcomes after mitral valve repair for IE are excellent, with 5-year freedom from reoperation exceeding 85% and 10-year freedom from reoperation of 80–85% [77, 78]. Predictors of repair failure include extensive leaflet destruction, anterior leaflet involvement, and S. aureus infection [77, 78]. For patients undergoing mitral valve replacement, 5-year survival ranges from 60 to 70%, with freedom from reoperation of 90–95% (lower reoperation rates than repair due to the definitive nature of replacement, but higher mortality) [8, 81].

## Right-sided valves: low-pressure protection and IVDU susceptibility

### Anatomical protection in low-pressure circulation

Native right-sided valves, the tricuspid and pulmonary valves, demonstrate markedly lower susceptibility to IE compared to left-sided valves, collectively accounting for only 5–10% of native valve IE cases [1, 3, 13]. This intrinsic protection is primarily attributable to the low-pressure hemodynamic environment of the right heart. The tricuspid valve operates with right ventricular systolic pressures of 25 mmHg and diastolic pressures of 0–8 mmHg, compared to left ventricular systolic pressures of 120 mmHg and diastolic pressures of 80 mmHg [33, 34]. The pulmonary valve experiences the lowest pressures in the cardiac cycle, with pulmonary artery systolic pressures of 25 mmHg and diastolic pressures of 8–12 mmHg [44, 47].

This low-pressure environment generates minimal shear stress on the valve endothelium, reducing the likelihood of endothelial injury and NBTE formation [15, 33]. Additionally, right-sided valves have simpler structural anatomy compared to left-sided valves: the tricuspid valve has three leaflets with less complex chordal architecture than the mitral valve, and the pulmonary valve is a semilunar valve similar to the aortic valve but with thinner leaflets and less fibrous tissue [14, 33]. The relative absence of calcific degeneration in right-sided valves (due to lower mechanical stress) further reduces substrate for bacterial adherence [13, 33].

### Tricuspid valve: IVDU-specific vulnerability

Despite the intrinsic anatomical protection of the tricuspid valve, it becomes the predominant site of IE in a specific high-risk population: intravenous drug users (IVDU). Tricuspid IE accounts for 60–90% of IE cases in IVDU, representing a complete reversal of the typical valve distribution [8, 9, 13, 32, 42, 43, 82–85]. This IVDU-specific vulnerability is driven by repeated bacteremia from contaminated injection equipment, direct endothelial trauma from particulate matter in injected substances, and the preferential deposition of bacteria in the right heart due to first-pass circulation [31, 35, 36].

S. aureus is the causative organism in 60–80% of IVDU-associated tricuspid IE, reflecting the high prevalence of S. aureus skin colonisation and the virulence of this pathogen [4, 36, 82]. The clinical presentation of tricuspid IE differs from left-sided IE: patients typically present with septic pulmonary emboli (causing pleuritic chest pain, cough, and hemoptysis) rather than systemic emboli, and heart failure is less common due to the right ventricle’s tolerance of volume overload [13, 35, 38].

### Surgical technique: repair, replacement, or valvectomy

Surgical management of tricuspid IE is guided by the principle that medical therapy alone is often sufficient for uncomplicated cases, with surgery reserved for refractory infection, severe tricuspid regurgitation with right heart failure, or large vegetations (> 20 mm) with recurrent septic emboli despite appropriate antibiotics [13, 39, 40]. When surgery is indicated, tricuspid valve repair or annuloplasty is strongly preferred over replacement, as the right ventricle tolerates moderate tricuspid regurgitation better than prosthetic valve dysfunction [40, 41, 81].

Repair techniques include vegetation debridement, leaflet repair with pericardial patch, chordal repair, and annuloplasty with a prosthetic ring or suture annuloplasty [40, 41, 81]. A systematic review and meta-analysis of tricuspid IE surgery reported 30-day mortality of approximately 10–15% for repair (a representative estimate from available series), with 5-year survival reported at approximately 60–65% in the available literature [81]. Tricuspid valve replacement, when necessary, is performed with bioprosthetic valves; mechanical valves are avoided in IVDU due to the high risk of reinfection and the challenges of anticoagulation compliance in this population [13, 39, 82].

Tricuspid valvectomy complete excision of the tricuspid valve without immediate replacement is a controversial option reserved for IVDU with active ongoing drug use, no reliable intravenous access for long-term antibiotics, and refractory infection [37, 42]. Valvectomy creates severe tricuspid regurgitation but allows the patient to survive the acute infection, with the option for delayed valve replacement once the infection is cleared and the patient has achieved sustained abstinence from drug use [37, 42]. Reported 30-day mortality after valvectomy is approximately 13%, with the understanding that this is a bridge procedure rather than a definitive treatment [39, 44].

### Clinical outcomes and IVDU-specific considerations

The 5-year survival after tricuspid valve surgery for IE is 63.9%, which is lower than mitral or aortic valve surgery, primarily due to the high-risk profile of the IVDU population and the substantial risk of reinfection [81, 82]. Ongoing intravenous drug use is the single strongest predictor of reinfection and reoperation, with reinfection rates of 20–40% at 5 years in patients who continue drug use [8, 9, 42, 43, 82–84]. In contrast, patients who achieve sustained abstinence have reinfection rates comparable to non-IVDU populations [82, 84].

Pulmonary valve IE is exceedingly rare, accounting for less than 2% of all IE cases [1, 43, 45]. When it occurs, it is typically in the setting of congenital heart disease (particularly tetralogy of Fallot with right ventricular outflow tract reconstruction), intravenous drug use, or the presence of pulmonary artery catheters [43, 45, 46]. Surgical management involves vegetation debridement and valve repair when possible, or pulmonary valve replacement with a pulmonary homograft for extensive destruction [46, 48].

## Prosthetic valve endocarditis: substrate-specific vulnerability

### Epidemiology and risk factors

Prosthetic valve endocarditis (PVE), encompassing infection of mechanical or bioprosthetic valves and repair materials, accounts for 10–30% of all IE cases [10, 11, 49]. Its incidence is approximately 0.3–1.2% per patient-year, with cumulative risk reaching 3–6% at 5 years, and it carries substantially higher morbidity and mortality than native valve IE [10, 49, 50].

PVE is classified as early (< 12 months post-surgery) or late (> 12 months), reflecting distinct pathogenic mechanisms: early PVE is typically nosocomial (CoNS, S. aureus, gram-negatives), while late PVE resembles native valve IE in microbiology (streptococci, enterococci) [10, 49, 51].

Transcatheter aortic valve implantation (TAVI) has introduced a new substrate for IE, with TAVI-associated IE (TAVI-IE) occurring at an incidence of 0.5-3% per patient-year [87]. TAVI-IE presents unique diagnostic and therapeutic challenges due to the difficulty of imaging the prosthesis with echocardiography and the technical complexity of explanting a transcatheter valve [57, 87].

### Anatomical patterns and imaging

The anatomical substrate of PVE differs fundamentally from native valve IE: the sewing ring is the primary infection site, where biofilm formation, bacteria embedded in a protective extracellular matrix, renders eradication with antimicrobial therapy alone unlikely, with cure rates below 30% [10, 49, 52, 54].

Peri-prosthetic dehiscence, the separation of the prosthetic valve from the native annulus due to infection-related tissue destruction, is a hallmark of PVE and results in severe peri-prosthetic regurgitation [10, 55]. Peri-annular abscess forms in 40–60% of PVE cases higher than the 30–40% rate in native aortic IE and may extend into the conduction system, aortomitral continuity, or ventricular myocardium, necessitating extensive reconstruction [7, 10, 55].

Imaging of PVE is challenging due to acoustic shadowing and reverberations from prosthetic material, which limit the sensitivity of transthoracic echocardiography (TTE) and even transesophageal echocardiography (TEE) [58, 61]. Cardiac computed tomography (CT) has emerged as superior to TEE for detecting peri-annular complications in PVE, with sensitivity of 96% for abscess detection compared to 50–70% for TEE [63, 64]. The 2023 ESC Guidelines recommend cardiac CT for all suspected PVE cases to assess the extent of peri-annular involvement and guide surgical planning [2, 63].

### Surgical technique and reconstruction challenges

Surgical management of PVE requires radical debridement of all infected tissue, including complete removal of the prosthetic valve, sewing ring, and any infected annular or peri-annular tissue [10, 11, 19–21, 56]. The extent of debridement often creates large annular defects that require complex reconstruction. For limited annular defects, pericardial or homograft patch repair can restore annular integrity and allow implantation of a new prosthetic valve [19, 21, 74].

For extensive peri-annular destruction in aortic PVE, cryopreserved aortic homograft (CAH) root replacement is preferred, offering resistance to reinfection and facilitating reconstruction of complex defects with viable tissue [19, 21, 71, 74]. The additional steps of prosthetic explantation and annular reconstruction are reflected in operative times approximately 60–70% longer than for native valve IE [56].

TAVI explantation for IE is technically demanding—the valve is often calcified and deeply embedded in the native annulus—with limited data reporting operative mortality of 20–30%, comparable to or exceeding conventional PVE surgery [57, 87].

### Clinical outcomes and re-surgery risk

Operative mortality for PVE ranges from 17 to 26%, substantially higher than the 8–15% reported for native valve IE [10, 11, 56]. In a representative single-centre series of 172 patients with aortic PVE, 30-day mortality was 17.4%, with multivalvular involvement conferring an OR of 8.04 for mortality [56]. Long-term survival is 72% at 1 year, 56% at 5 years, and 46% at 10 years [56].

Freedom from reoperation is approximately 91% at 10 years; redo AVR for recurrent PVE or structural deterioration carries 30-day mortality of 15–21% [86]. Independent predictors of mortality include S. aureus infection (OR 4.2), heart failure (OR 3.1), peri-annular abscess (OR 2.3), and renal failure (OR 2.8) [10, 11, 56].

## Multimodality imaging for structural assessment

### Transoesophageal echocardiography (TEE)

Transoesophageal echocardiography (TEE) remains the gold standard imaging modality for IE, with sensitivity of 87–100% for vegetation detection and superior visualisation of valve morphology, leaflet perforation, and chordal rupture compared to transthoracic echocardiography (TTE) [28, 29, 58, 60, 61]. TEE is essential for surgical planning, providing detailed assessment of vegetation size and location, extent of leaflet destruction, presence of abscess, and involvement of the subvalvular apparatus [58, 60]. The 2015 ESC Guidelines recommend TEE for all patients with suspected IE, with repeat TEE if initial imaging is negative but clinical suspicion remains high [1, 58].

Real-time three-dimensional (3D) TEE has further enhanced visualisation of complex valve anatomy, particularly for mitral valve IE, allowing precise localisation of perforations and assessment of repair feasibility [62, 69]. 3D TEE enables en face views of the mitral valve from both atrial and ventricular perspectives, facilitating surgical planning for leaflet repair and annuloplasty [62]. Vegetation size measured by TEE correlates with embolic risk: vegetations greater than 10 mm are associated with increased risk of systemic embolism, and vegetations greater than 15 mm are associated with higher mortality [24, 27, 69].

### Cardiac Computed Tomography (CT)

Cardiac CT has emerged as a critical complementary imaging modality to TEE, particularly for assessment of peri-annular complications [63–65]. CT demonstrates superior sensitivity for abscess detection compared to TEE (96% versus 50–70%), due to its ability to visualise the entire aortic root and peri-annular structures without acoustic shadowing [63, 64]. CT is particularly valuable in prosthetic valve endocarditis (PVE), where prosthetic material limits TEE visualisation [63, 66].

Three-dimensional CT reconstruction allows detailed surgical planning, including measurement of annular dimensions, assessment of the spatial relationship between abscesses and adjacent structures (coronary ostia, conduction system, aortomitral continuity), and identification of aorto-cavitary fistulae [63, 64]. The 2023 ESC Guidelines recommend cardiac CT for all patients with suspected peri-annular extension and as a mandatory preoperative imaging modality for surgical planning [2, 63]. CT angiography also enables assessment of coronary artery anatomy, which is essential for planning aortic root replacement with coronary reimplantation [63, 64].

### Nuclear imaging (PET/CT and WBC-SPECT/CT)

Nuclear imaging with 18 F-fluorodeoxyglucose positron emission tomography/computed tomography (18 F-FDG PET/CT) and white blood cell single-photon emission computed tomography/computed tomography (WBC-SPECT/CT) has gained increasing recognition for diagnosis of IE, particularly in challenging cases such as PVE and TAVI-IE [59, 65, 67, 68]. 18 F-FDG PET/CT detects increased metabolic activity in infected tissue, with sensitivity of 73% and specificity of 80% for PVE diagnosis [65, 67]. PET/CT is particularly valuable for early PVE (within 3 months of surgery), where TEE sensitivity is limited due to postoperative changes [59, 67].

The 2023 ESC Guidelines have incorporated PET/CT and WBC-SPECT/CT into the diagnostic criteria for IE, recognising abnormal uptake around a prosthetic valve as a major criterion for PVE diagnosis [2, 59, 67]. Nuclear imaging also enables detection of embolic complications and metastatic infection, providing whole-body assessment that complements cardiac-focused imaging [67, 68]. This vulnerability hierarchy across valve types, integrates these imaging findings with anatomical determinants to guide risk stratification and surgical planning.

## Surgical timing and decision-making

### ESC guidelines 2023 surgical indications

The 2023 ESC Guidelines for the management of IE provide a comprehensive framework for surgical timing, categorising indications into emergency (surgery within 24 h), urgent (surgery within 7 days), and elective (surgery after completion of antibiotic therapy or at optimal timing) [2]. Emergency surgery is indicated for refractory cardiogenic shock or pulmonary edema despite optimal medical therapy, reflecting hemodynamic instability that cannot be stabilised with inotropes and mechanical support [2, 9]. Urgent surgery is indicated for three primary scenarios: heart failure due to severe valve regurgitation or obstruction, uncontrolled infection (persistent bacteremia despite appropriate antibiotics, abscess formation, or fistula), and prevention of embolism in patients with large vegetations (> 10 mm) and high embolic risk [2, 9].

The landmark EASE trial (Early Surgery versus Conventional Treatment for Infective Endocarditis) demonstrated that early surgery (within 48 h of randomisation) significantly reduced the composite endpoint of death and embolic events compared to conventional treatment (3% versus 23%, *p* = 0.001) in patients with large vegetations (> 10 mm) and severe valve dysfunction [9]. This trial established early surgery as a Class I recommendation for patients with large vegetations and embolic risk, fundamentally changing practice patterns [1, 2, 9].

### Valve-specific decision framework

Surgical decision-making in IE must integrate guideline-based indications with valve-specific anatomical considerations. For aortic valve IE, the presence of peri-annular extension (abscess, fistula, or conduction disturbances) mandates urgent surgery, as medical therapy alone cannot eradicate infection in the fibrous skeleton [6, 7, 71, 72]. The extent of peri-annular destruction determines the surgical technique: localised abscesses may be managed with patch repair and valve replacement, whereas extensive destruction involving the intervalvular fibrous body requires root replacement with homograft or complex reconstruction [70–72].

For mitral valve IE, the feasibility of repair is a critical determinant of surgical timing and approach [8, 30, 77–79]. When repair is feasible, early surgery is preferred to prevent progressive leaflet destruction that may preclude repair [8, 77]. Preoperative assessment with TEE and 3D echocardiography is essential to determine repair feasibility, with intraoperative assessment by an experienced surgeon providing the final determination [62, 77, 78]. The presence of annular abscess does not preclude repair if adequate debridement and reconstruction can be achieved [7, 30].

For tricuspid valve IE, particularly in IVDU, medical therapy is often attempted first, with surgery reserved for refractory infection, severe right heart failure, or recurrent septic emboli despite appropriate antibiotics [13, 39, 40, 81]. The decision to perform repair, replacement, or valvectomy depends on the extent of destruction, the patient’s drug use status, and the likelihood of compliance with postoperative care [37, 39, 42, 82].

For PVE, early surgery is associated with improved outcomes compared to prolonged medical therapy, as biofilm-associated infection is rarely eradicated with antibiotics alone [10, 11, 88]. The 2023 ESC Guidelines recommend urgent surgery for PVE with heart failure, uncontrolled infection, or peri-annular extension [2, 10, 11].

### Neurological considerations

Neurological complications, particularly cerebral embolism, occur in 20–40% of IE cases and represent a major determinant of surgical timing [89]. The risk of hemorrhagic transformation of ischemic stroke is highest in the first 48–72 h after embolism, and cardiac surgery with cardiopulmonary bypass and anticoagulation during this period carries substantial risk of catastrophic intracranial hemorrhage [89]. Current guidelines recommend delaying surgery for 2–4 weeks after major ischemic stroke (with neurological deficit) to allow stabilisation of the infarct and reduce hemorrhagic risk [2, 89].

However, minor stroke or transient ischemic attack (TIA) without significant neurological deficit is not a contraindication to early surgery, and the embolic risk of delaying surgery may outweigh the hemorrhagic risk [2, 89]. Silent cerebral emboli, detected on brain magnetic resonance imaging (MRI) in up to 50% of IE patients, do not alter surgical timing [89]. The decision to proceed with surgery in the setting of neurological complications requires multidisciplinary discussion involving cardiology, cardiac surgery, neurology, and infectious diseases [2, 89].

## Structural vulnerability impact on surgical outcomes

### Operative complexity hierarchy

The proposed structural vulnerability gradient, prosthetic valves > aortic > mitral > tricuspid > pulmonary (native), appears to correlate with operative complexity, as quantified by cardiopulmonary bypass (CPB) times, cross-clamp times, and perioperative mortality (Tables 1, 2 and 3). Aortic valve IE with peri-annular extension represents the most complex native valve surgery, with homograft root replacement requiring substantially prolonged CPB and cross-clamp times (representative published estimates: approximately 190–250 and 140–180 min, respectively) [71]. Intervalvular fibrous body (IFB) reconstruction for extensive aortic-mitral IE requires markedly prolonged CPB times, reflecting the technical demands of radical debridement and complex reconstruction [72].

**Table 1:** Comparative Structural Vulnerability Across Cardiac Valves in Infective Endocarditis. Vulnerability grade represents a conceptual synthesis of anatomical susceptibility, haemodynamic stress, propensity for peri-annular extension, and anticipated surgical complexity. It is intended as a qualitative framework rather than a validated prognostic score

Valve type	Anatomical risk factors	Haemodynamic mechanism	Infection pattern	Extension risk	Vulnerability grade
Aortic (Native)	Fibrous skeleton continuity; age-related calcification; bicuspid aortic valve (1–2% prevalence)	Sustained high shear stress (systolic pressure ~ 120 mmHg); turbulent flow across calcified leaflets	Leaflet vegetation; peri-annular abscess (30–40%); aorto-cavitary fistula (1.6–3.2%)	High: intervalvular fibrous body, mitral valve, conduction system, ventricular septum	**Very High**
Mitral (Native)	Aortomitral continuity; mitral valve prolapse (2–3% prevalence); rheumatic valve disease; regurgitant jet lesions	Conditional vulnerability requiring pre-existing structural abnormality; regurgitant jet impact on atrial leaflet surface	Leaflet perforation; chordal rupture; annular abscess (10–15%); extension from aortic IE	Moderate: aortomitral continuity and left atrial wall	**Moderate–High**
**Tricuspid (Native)**	Relative anatomical isolation from fibrous skeleton; simpler chordal architecture	Low-pressure environment (right atrial pressure 0–8 mmHg); minimal shear stress	Predominantly associated with intravenous drug use; repeated bacteraemia; *Staphylococcus aureus* (60–80%); septic pulmonary emboli	Low: extension beyond the valve apparatus is uncommon	**Low** *(High in IVDU-associated IE)*
Pulmonary (Native)	Lowest-pressure valve in the cardiac cycle; minimal calcification; simple semilunar structure	Pulmonary artery pressure ~ 25/8–12 mmHg; minimal endothelial injury	Rare; associated with congenital heart disease, intravenous drug use, or pulmonary artery catheters; <2% of all IE cases	Very low: usually confined to the valve	**Very Low**
Prosthetic (Any Position)	Sewing-ring biofilm formation; peri-prosthetic dehiscence; avascular prosthetic material	Biofilm-mediated infection with impaired antimicrobial penetration; early (nosocomial) versus late (community-acquired) PVE	Sewing-ring infection; peri-annular abscess (40–60%); dehiscence causing severe regurgitation	Very high: extensive peri-annular destruction, fistula formation, annular disruption	**Highest**

IFB, intervalvular fibrous body; IE, infective endocarditis; IVDU, intravenous drug user; PVE, prosthetic valve endocarditis

**Table 2:** Surgical Technique Selection According to Extent of Structural Destruction in Infective Endocarditis. Surgical technique selection is influenced by the extent of structural destruction, peri-annular involvement, patient-specific factors, causative organism, and institutional expertise. Recommendations represent commonly reported approaches within contemporary surgical series and should not be interpreted as prescriptive treatment algorithms

Valve type	Extent of structural destruction	Preferred surgical technique	Alternative surgical technique	Special considerations
Aortic (Localised Disease)	Leaflet vegetation without peri-annular extension	Aortic valve replacement (bioprosthetic or mechanical)	Leaflet repair with pericardial patch (rarely feasible)	Prosthesis selection remains debated; some centres favour bioprosthetic valves during active infection
Aortic (Peri-annular Disease)	Abscess confined to the annulus without intervalvular fibrous body involvement	Aortic valve replacement with patch reconstruction of the abscess cavity (pericardial or homograft patch)	Homograft root replacement	Patch reconstruction may be feasible when annular integrity is preserved and the abscess cavity is limited
Aortic (Extensive Disease / IFB Involvement)	Intervalvular fibrous body destruction, aorto-cavitary fistula, or extensive peri-annular abscess	Homograft root replacement, Bentall procedure, or Commando procedure	Prosthetic composite graft with patch reconstruction	Homografts may offer resistance to reinfection; Commando procedure is reserved for extensive intervalvular fibrous body involvement
Mitral (Leaflet-Limited Disease)	Leaflet perforation, chordal rupture, without annular involvement	Mitral valve repair (pericardial patch reconstruction, chordal replacement, annuloplasty)	Mitral valve replacement (usually bioprosthetic)	Repair is preferred whenever feasible and is associated with lower early mortality compared with replacement
Mitral (Annular Disease)	Annular abscess or extensive leaflet destruction	Mitral valve replacement with annular debridement and reconstruction	Mitral valve repair with patch reconstruction in selected cases	Bioprosthetic valves are frequently preferred in active infection
Tricuspid (Vegetation-Predominant Disease)	Vegetation without extensive leaflet destruction	Initial medical therapy; surgery when indicated using repair or annuloplasty	Tricuspid valve replacement (bioprosthetic)	Surgery is generally reserved for refractory infection, severe tricuspid regurgitation with right ventricular failure, or recurrent septic pulmonary emboli
Tricuspid (IVDU-Associated Extensive Disease)	Extensive destruction in active intravenous drug users with ongoing high reinfection risk	Tricuspid valvectomy as a bridge strategy	Valve repair when feasible and abstinence is achievable	Delayed valve replacement may be considered after infection eradication and sustained abstinence
Prosthetic Valve Endocarditis (Limited Disease)	Sewing-ring infection without extensive peri-annular destruction	Redo valve replacement with patch reconstruction of limited defects	Homograft root replacement	Complete removal of prosthetic material is mandatory
Prosthetic Valve Endocarditis (Extensive Disease)	Peri-annular abscess, prosthetic dehiscence, or fistula formation	Cryopreserved aortic homograft root replacement	Prosthetic composite graft with extensive patch reconstruction	Homograft root replacement is often preferred for extensive destruction; TAVI explantation remains technically demanding

AVR, aortic valve replacement; CAH, cryopreserved aortic homograft; IFB, intervalvular fibrous body; IVDU, intravenous drug user; PVE, prosthetic valve endocarditis; TAVI, transcatheter aortic valve implantation

**Table 3:** Perioperative Outcomes According to Valve Type and Surgical Approach in Infective Endocarditis. Values are reported as mean ± standard deviation, mean values, pooled estimates, or ranges derived from the cited studies. Mortality estimates are based predominantly on observational cohort studies and meta-analyses. Freedom-from-reoperation estimates for tricuspid valve surgery exclude patients with ongoing intravenous drug use, in whom reinfection rates may reach 20–40% within 5 years. Outcomes should be interpreted in the context of substantial heterogeneity in patient characteristics, operative eras, and surgical techniques across studies

Valve/Procedure	Cardiopulmonary bypass time (min)	Cross-clamp time (min)	30-day mortality (%)	5-year survival (%)	10-year freedom from reoperation (%)	Key references
Aortic Homograft Root Replacement	214 ± 80	164 ± 56	18–22	58–65	85–90	[21, 74]
Aortic Commando Procedure / IFB Reconstruction	236.9 ± 11.1	Not reported	28.3 *(90-day mortality: 37.0%)*	Not reported	Not reported	[72]
Mitral Valve Repair	90–120	60–90	3.7 *(pooled estimate)*	75–80	80–85	[8, 77, 78]
Mitral Valve Replacement	100–130	70–100	10.9 *(pooled estimate)*	60–70	90–95	[8, 81]
Tricuspid Valve Repair	60–90	40–60	12.5	63.9	> 90 *(non-IVDU populations)*	[81]
Tricuspid Valvectomy	40–60	30–45	13	Not reported *(bridge procedure)*	Not applicable	[39, 44]
Prosthetic Valve Endocarditis Surgery (Any Valve Position)	166 *(mean)*	Not reported	17–26	56	~ 91	[10, 11, 56, 86]
Redo Aortic Valve Replacement (Recurrent PVE or Structural Valve Deterioration)	Not reported	Not reported	15–21	Not reported	Not applicable	[86]

IFB, intervalvular fibrous body; IVDU, intravenous drug user; PVE, prosthetic valve endocarditis

Prosthetic valve endocarditis (PVE) surgery demonstrates the highest operative complexity among all IE subtypes, with substantially prolonged CPB and operative times compared to native valve IE, reflecting the additional steps of prosthetic explantation and annular reconstruction [56]. This prolonged operative time reflects the need for complete removal of prosthetic material, extensive debridement of infected tissue, and complex reconstruction of annular defects [56, 74]. Mitral valve repair for IE, when feasible, requires moderate CPB times but demands meticulous surgical technique to achieve durable repair [8, 77, 78].

Tricuspid valve surgery for IE demonstrates comparatively shorter CPB times due to the relative simplicity of right-sided valve surgery and the lower technical demands of tricuspid repair compared to mitral repair [40, 81]. However, the overall complexity of tricuspid IE management is increased by the high-risk patient population (IVDU) and the substantial risk of reinfection [82–84].

### Predictors of surgical mortality

Multivariate analyses across multiple large cohort studies have identified consistent independent predictors of surgical mortality in IE [12, 39, 56, 71, 75, 77]. Pathogen virulence is a dominant predictor: S. aureus IE carries odds ratios of 3.2–4.8 for mortality compared to streptococcal IE, reflecting the aggressive tissue destruction, high rate of metastatic infection, and frequent association with septic shock characteristic of S. aureus [4, 12, 71, 75]. Fungal IE, though rare, carries even higher mortality (> 50%) due to the difficulty of eradicating fungal biofilms and the limited efficacy of antifungal therapy [53].

Extent of structural destruction, particularly peri-annular abscess, approximately doubles mortality risk (OR 2.1–2.3) compared to IE confined to valve leaflets [7, 12, 71, 75]. This reflects both the technical complexity of reconstruction and the systemic inflammatory burden associated with extensive infection [7, 71]. Cardiac function, quantified by left ventricular ejection fraction (EF), is a powerful predictor: EF less than 35% confers odds ratios of 2.5–3.5 for mortality, reflecting the inability of the failing ventricle to tolerate the hemodynamic stress of cardiopulmonary bypass and the postoperative period [12, 75, 77].

Reoperation for IE (surgery for PVE or recurrent native valve IE) carries 30-day mortality of 15–21% compared to 8–12% for primary surgery, reflecting the technical challenges of reoperative surgery, the presence of prosthetic material or scar tissue, and the often more virulent organisms (S. aureus, CoNS) associated with reinfection [10, 11, 56, 86]. Emergency surgery, defined as surgery within 24 h due to hemodynamic instability, carries odds ratios of 2.5-3.0 for mortality compared to urgent or elective surgery [12, 39, 75].

### Re-surgery risk and long-term outcomes

Freedom from reoperation is a critical long-term outcome metric in IE surgery, reflecting both the durability of the initial repair or replacement and the risk of reinfection. For aortic valve surgery, 10-year freedom from reoperation ranges from 85 to 90%, with homograft root replacement demonstrating similar or slightly lower reoperation rates compared to prosthetic valve replacement [71, 74, 86]. Mitral valve repair demonstrates 10-year freedom from reoperation of 80–85%, with repair failure typically occurring within the first 2 years due to recurrent infection or inadequate initial repair [77, 78].

Tricuspid valve surgery demonstrates highly variable reoperation rates depending on the patient population: in non-IVDU patients, 10-year freedom from reoperation exceeds 90%, whereas in IVDU with ongoing drug use, reoperation rates approach 30–40% at 5 years due to reinfection [81–84]. This stark difference underscores the importance of addressing the underlying risk factor (intravenous drug use) to achieve durable surgical outcomes [82, 84].

Prosthetic valve endocarditis (PVE) surgery demonstrates 10-year freedom from reoperation of approximately 85–91% across published series, indicating that most patients who survive the initial operation do not require further valve surgery [86]. However, when reoperation is required for recurrent PVE or structural valve deterioration, mortality is substantial (15–21% at 30 days), emphasising the importance of achieving complete eradication of infection at the initial operation [86]. Predictors of reoperation include ongoing IVDU, mechanical valve prosthesis (higher reinfection risk than bioprosthesis), young age (longer exposure time to reinfection risk), and S. aureus as the initial causative organism [82, 84, 86].

## Critical synthesis: controversies, competing paradigms, and the structural vulnerability framework

The proposed vulnerability hierarchy, prosthetic valves > aortic > mitral > tricuspid > pulmonary (native), is not merely a descriptive taxonomy but a mechanistically grounded conceptual model that organises the complex interplay of anatomy, hemodynamics, microbiology, and surgical technique in IE. However, several areas of genuine clinical controversy deserve critical appraisal, as the existing literature is characterised by significant methodological heterogeneity, publication bias, and unresolved mechanistic questions. The following controversies directly bear on the framework’s clinical applicability and highlight where the evidence base requires strengthening.

### Homograft versus prosthesis in aortic IE: a contested superiority

The use of cryopreserved aortic homografts (CAH) for aortic root replacement in active IE has long been advocated on the basis of theoretical resistance to reinfection, the ability to replace infected tissue with viable biological material, and facilitation of complex reconstruction in the setting of annular destruction [71, 72]. However, the evidence base for homograft superiority over prosthetic alternatives is substantially weaker than is commonly acknowledged, and the framework must be applied with this uncertainty in mind.

The landmark series from Yankah et al. [21] and Sabik et al. [74] demonstrated acceptable long-term outcomes with homograft root replacement (10-year survival 58–65%; freedom from reoperation 85–90%), yet both were single-centre, non-randomised, and subject to selection bias favouring homografts in younger patients with more extensive destruction [21, 74]. Perrotta et al. found no statistically significant difference in early mortality, late survival, or freedom from reoperation between homograft and prosthetic root replacement [56]—a finding corroborated by STS registry data [75]—and Avierinos et al. concluded that outcomes are determined by the extent of debridement rather than prosthesis choice [73].

Practical limitations of homografts, limited availability, progressive structural deterioration, and technical demands restricting use to high-volume centres, reinforce an individualised approach [71, 72, 74]. This review contextualises homograft use for cases with extensive annular destruction, IFB involvement, or aorto-cavitary fistulae; prosthetic composite grafts remain appropriate for localised to moderate aortic IE, with conduit choice guided by extent of destruction, patient age, and centre expertise.

## Repair versus replacement bias in mitral IE: confounding and causation

The consistent finding that mitral valve repair is associated with significantly lower early mortality than replacement (3.7% vs. 10.9%; HR 0.70, 95% CI 0.55–0.89) has been interpreted as evidence of the superiority of repair as a surgical technique [8, 77, 78]. This interpretation requires critical scrutiny: repair is performed preferentially in patients with less extensive destruction and more favourable anatomy, while replacement is reserved for advanced disease, annular involvement, or prior valve surgery [77, 78]—a selection effect that substantially confounds the apparent technical advantage.

In the meta-analysis by Feringa et al. (*n* = 1,076), pooled early mortality was 3.7% for repair versus 10.9% for replacement (adjusted HR 0.70, 95% CI 0.55–0.89) [8]; however, incomplete case-mix adjustment and study heterogeneity limit causal inference. The repair advantage likely reflects both genuine technical benefit preservation of subvalvular apparatus, avoidance of prosthetic material in an infected field and a selection effect that cannot be fully disentangled from retrospective data.

The framework predicts that mitral IE, by virtue of its conditional vulnerability, is more amenable to repair than aortic IE precisely because structural destruction is typically less extensive. However, this prediction should not translate into an uncritical pursuit of repair at the expense of complete infection eradication. The development of validated intraoperative criteria for repair feasibility in IE, analogous to degenerative mitral repair scoring systems remains an unmet need and represents a priority for future research.

### TAVI-associated IE: an emerging substrate challenging the gradient

Transcatheter aortic valve implantation (TAVI) has transformed the management of aortic stenosis, with exponential global growth in procedural volume since its introduction [2, 57]. The emergence of TAVI-associated IE (TAVI-IE) represents a new and poorly characterised substrate that challenges the analytical framework developed from surgical valve data. The incidence of TAVI-IE ranges from 0.5% to 3.1% per patient-year, with early TAVI-IE (within 12 months) predominantly caused by S. aureus and Enterococcus spp., reflecting a nosocomial origin related to vascular access and permanent pacemaker implantation [87].

Unlike surgical bioprosthetic PVE, the transcatheter prosthesis is deployed without annular debridement, creating anatomical recesses inaccessible to antibiotic therapy; peri-prosthetic regurgitation in up to 20% of first-generation devices further predisposes to vegetation formation [57, 87].

The current hierarchy places TAVI-IE within the PVE category, but the distinct anatomical substrate, the higher-risk patient population, and the unique explantation challenges suggest that TAVI-IE may warrant its own category in future iterations of the model. Surgical management of TAVI-IE is technically demanding and poorly standardised, with explantation associated with 30–50% mortality in small series [87, 88]. Prospective registries with standardised diagnostic criteria and long-term follow-up are urgently needed.

### Imaging limitations and the structural vulnerability assessment gap

Accurate preoperative characterisation of structural vulnerability, the foundation of the gradient framework depends critically on multimodality imaging, but each modality has important limitations that constrain the precision of preoperative planning.

TEE, the primary diagnostic tool, achieves sensitivity of 87–100% for vegetations > 10 mm but only 40–60% for early peri-annular abscess, with acoustic shadowing further limiting assessment in PVE [28, 29]. Cardiac CT (sensitivity 80–96% for peri-annular complications) overcomes these limitations for surgical planning, while 18 F-FDG PET/CT (sensitivity 73–93%) serves primarily a diagnostic role for PVE and TAVI-IE [30–33]. Critically, no modality can reliably predict intraoperative tissue quality, the key determinant of repair feasibility in mitral IE underscoring the irreplaceable role of surgical judgment and the limits of any preoperative vulnerability assessment.

## Limitations of current evidence

### Study design heterogeneity and the absence of randomised data

The evidence base for surgical management of IE is characterised by a near-total absence of randomised controlled trial (RCT) data. The ethical and logistical challenges of randomising patients with active, life-threatening infection to different surgical strategies are substantial, and large-scale RCTs are unlikely to be conducted in the foreseeable future. As a consequence, the surgical literature consists predominantly of retrospective single-centre cohort studies, with a smaller number of multicentre registries and systematic reviews of observational data [1, 2, 12].

This observational evidence base is subject to multiple forms of bias such as selection bias where patients too sick for surgery are excluded, ascertainment bias which is minor complications inconsistently documented, and survivorship bias where long-term follow-up available only for a selected subset of hospital survivors.

The framework synthesised in this review is therefore built on a foundation of observational data with moderate-to-high risk of bias. The mortality figures cited aortic IE 18–28%, PVE 17–26%, mitral repair 3.7% vs. replacement 10.9% should be interpreted as estimates with wide confidence intervals reflecting the heterogeneity of underlying populations, operative eras, and surgical techniques, rather than as precise point estimates applicable to individual patients.

### Outcome definition heterogeneity

A fundamental challenge in synthesising IE surgical outcomes is the inconsistent definition of key endpoints across studies. “Operative mortality” is variously defined as in-hospital death, 30-day mortality, or death within 30 days of surgery regardless of discharge status; these definitions yield substantially different rates in high-risk patients with prolonged ICU stays. “Peri-annular abscess” is defined anatomically in some studies (suppurative cavity > 5 mm on imaging) and operatively in others (intraoperative finding of pus or cavitation), with significant implications for reported prevalence (15–60% across studies [6, 7, 10]). “Repair” versus “replacement” is inconsistently defined, with some studies classifying pericardial patch repair of leaflet perforations as “repair” and others as “replacement with autologous tissue.”

This definitional heterogeneity is particularly problematic for the meta-analytic data on mitral repair versus replacement, where pooled estimates combine studies with substantially different definitions of repair, thresholds for repair feasibility, and operative eras spanning three decades of evolving surgical technique contributing to the controversy discussed in Sect.  11.2.

### Publication bias and centre volume effects

The IE surgical literature is dominated by reports from high-volume centres with established expertise in complex valve reconstruction, homograft implantation, and reoperative surgery. STS database analyses demonstrate that 30-day mortality for IE surgery varies from 8% to 35% across centres, with significantly lower mortality at centres performing > 20 IE operations per year [12]. The majority of published series originate from centres in the upper quartile of volume, introducing a systematic bias toward more favourable outcomes than would be observed in population-level data. Publication bias further distorts the literature, positive findings, repair feasibility, homograft durability, successful complex reconstruction are more likely to be published than negative findings, limiting the ability to draw firm conclusions about the boundaries of the framework.

### Temporal heterogeneity and evolving surgical practice

The IE surgical literature spans more than four decades, during which operative techniques, prosthetic valve technology, antibiotic regimens, and perioperative management have evolved substantially. Studies from the 1990s and early 2000s report outcomes with first-generation bioprostheses, limited intraoperative imaging, and pre-ERAS perioperative protocols that are not directly comparable to contemporary practice.

The widespread adoption of intraoperative TEE (mid-1990s), cardiac CT for preoperative planning (2000s), and 18 F-FDG PET/CT for PVE diagnosis (2010s) has transformed the precision of structural assessment and surgical planning. This model is more applicable to contemporary practice than to historical series, and the outcome data cited should be interpreted in the context of imaging technology available at the time of each study.

### Generalisability limitations

The majority of published surgical series originate from Europe and North America, with limited data from Asia, Africa, and Latin America, where the microbial epidemiology (higher prevalence of streptococcal IE, rheumatic valve disease as a risk factor), healthcare infrastructure, and surgical expertise differ substantially. The vulnerability hierarchy may not be directly applicable to populations with a high prevalence of rheumatic mitral disease, where the anatomical substrate of mitral IE differs fundamentally from the degenerative disease predominant in Western series.

Additionally, the growing population of elderly patients (> 75 years) with multiple comorbidities and frailty increasingly important in IE is underrepresented in surgical series. The risk-benefit calculus in frail elderly patients involves functional status, cognitive reserve, and patient preferences that the framework does not fully capture.

## Evidence gaps and future directions

### Unresolved questions in the structural vulnerability framework

The proposed structural vulnerability gradient, prosthetic valves > aortic > mitral > tricuspid > pulmonary (native) is a conceptual framework grounded in anatomical, hemodynamic, and surgical outcome data. However, several fundamental questions remain unanswered that limit its clinical applicability and scientific precision.

What is the relative contribution of anatomical versus microbial factors to valve-specific vulnerability? The gradient assigns primacy to anatomical determinants, but S. aureus demonstrates a different distribution pattern than streptococcal IE with higher rates of tricuspid and prosthetic valve involvement requiring prospective studies with detailed microbiological and anatomical phenotyping to disentangle these contributions.

Can this framework be operationalised as a validated preoperative scoring system? The current framework is qualitative. A validated quantitative score incorporating valve type, extent of peri-annular destruction (graded by CT), causative organism, and cardiac function would have direct clinical utility for risk stratification, surgical planning, and prognostic counselling. Development and validation of such a score would require multicentre prospective data collection with standardised imaging protocols and outcome definitions.

What is the optimal prosthesis choice for aortic IE in the contemporary era? The homograft versus prosthesis controversy (Sect.  11.1) remains unresolved; a multicentre registry with propensity-score matching and ≥ 10-year follow-up is required.

### TAVI-IE: an urgent research priority

The expansion of TAVI to younger, lower-risk patients increases the lifetime burden of TAVI-IE. Key unanswered questions anatomical predictors of TAVI-IE, the role of antibiotic prophylaxis, criteria for conservative versus surgical management, and long-term outcomes of explantation require prospective registries with mandatory reporting, standardised diagnostic criteria, and long-term follow-up to integrate TAVI-IE into this framework.

### Repair feasibility criteria and intraoperative decision-making

The absence of validated intraoperative criteria for mitral valve repair feasibility in IE represents a major evidence gap. Current practice relies on individual surgeon experience and judgment, leading to substantial inter-centre variability in repair rates (20–80% across published series [29–31]). A prospective study using standardised intraoperative assessment criteria incorporating leaflet tissue quality, annular integrity, chordal involvement, and extent of active infection and correlating these criteria with repair durability and outcomes would provide the evidence base for a standardised repair feasibility algorithm applicable across centres.

### Outcome registries and standardised complexity metrics

The development of valve-specific IE outcome registries with mandatory anatomical data collection represents the most important infrastructure investment for advancing the field. Such registries should capture: (1) preoperative imaging data with standardised grading of destruction extent; (2) intraoperative surgical complexity metrics (CPB time, cross-clamp time, number of reconstructive procedures); (3) short-term outcomes (30-day mortality, major adverse cardiac events, stroke, renal failure); (4) long-term outcomes (5- and 10-year survival, freedom from reoperation, valve-related complications); and (5) patient-reported outcomes. The Euro Heart Survey on IE and the International Collaboration on Endocarditis (ICE) registry have provided foundational epidemiological data, but surgical complexity and long-term outcomes remain incompletely captured.

### Addressing social determinants: IVDU and IE

The vulnerability concept developed here identifies IVDU as a modifier that transforms the tricuspid valve from the lowest-vulnerability native valve to a high-vulnerability substrate. The management of IVDU-associated IE is increasingly recognised as a complex sociomedical problem that cannot be addressed by surgical technique alone. Evidence-based addiction treatment (medication-assisted treatment with buprenorphine or methadone), harm reduction programmes, and integrated care models combining infectious disease, cardiothoracic surgery, and addiction medicine have demonstrated promising results in reducing reinfection rates and improving long-term outcomes [82, 84]. Future research should evaluate the cost-effectiveness of integrated care models for IVDU-IE and identify the surgical and social predictors of sustained abstinence and durable surgical outcomes.

## Conclusions

Infective endocarditis is best understood as a valve- and substrate-specific disease in which anatomical determinants hemodynamic stress, fibrous cardiac skeleton architecture, tissue composition, and spatial relationships fundamentally drive disease susceptibility, patterns of destruction, surgical complexity, and clinical outcomes. This narrative review proposes a structural vulnerability gradient prosthetic valves > aortic > mitral > tricuspid > pulmonary (native) as a conceptual model that appears to correlate with operative complexity, perioperative mortality, and long-term outcomes across published series.

The aortic valve demonstrates maximal intrinsic vulnerability due to sustained high shear stress, fibrous skeleton continuity enabling peri-annular extension, and propensity for complex complications including abscess formation, aorto-cavitary fistulae, and conduction disturbances. Surgical management of aortic IE ranges from straightforward valve replacement for localised disease to radical root reconstruction with homograft or complex intervalvular fibrous body repair, with 30-day mortality ranging from 8% to 37% depending on the extent of destruction [12, 71, 72, 75].

The mitral valve exhibits conditional vulnerability, typically requiring pre-existing structural abnormalities for infection to occur. The feasibility of mitral valve repair is the dominant determinant of outcomes, with repair conferring a 66% reduction in early mortality compared to replacement (3.7% versus 10.9%) and significantly improved long-term survival (adjusted HR 0.70) [29–31]. This establishes repair as the gold standard when technically feasible, with extensive preoperative imaging and intraoperative assessment essential for determining repair feasibility.

Native right-sided valves are intrinsically protected by low-pressure hemodynamics, but the tricuspid valve becomes highly vulnerable in the specific context of intravenous drug use, accounting for 60–90% of IE cases in this population [8, 9, 42, 43]. Surgical outcomes are heavily influenced by ongoing drug use, with reinfection rates of 20–40% at 5 years in patients who continue injection drug use, compared to rates comparable to non-IVDU populations in those who achieve sustained abstinence [8, 9, 42, 43].

Prosthetic valve endocarditis represents the highest-risk substrate, with operative mortality reported across series in the range of approximately 17–26% (a representative estimate from heterogeneous observational data), prolonged operative times, and substantial technical complexity due to the need for complete removal of prosthetic material and reconstruction of annular defects. Early surgery is associated with improved outcomes compared to prolonged medical therapy, as biofilm-associated infection is rarely eradicated with antibiotics alone.

The clinical implications of this valve-specific framework are substantial. Preoperative imaging with multimodality assessment (TEE, cardiac CT, and nuclear imaging for PVE) enables precise characterisation of the extent of destruction and guides surgical planning. Risk stratification based on valve type, extent of peri-annular involvement, causative organism, and cardiac function allows for informed prognostic counselling and optimisation of perioperative management. Surgical decision-making must integrate guideline-based indications with valve-specific anatomical considerations, recognising that the feasibility of repair (particularly for mitral IE), the extent of peri-annular destruction (particularly for aortic IE), and the patient’s drug use status (for tricuspid IE) are critical determinants of surgical approach and outcomes.

Future research directions should focus on establishing valve-specific outcome registries that capture detailed anatomical data, surgical techniques, and long-term outcomes to refine risk prediction models. Standardised complexity metrics that quantify the extent of structural destruction and the technical demands of reconstruction would facilitate comparison across centres and enable quality improvement initiatives. The integration of advanced imaging modalities, including 3D echocardiography, cardiac CT, and PET/CT, into routine preoperative assessment will continue to enhance surgical planning and improve outcomes. Finally, addressing the social determinants of health that drive the IVDU epidemic is essential to reducing the burden of tricuspid IE and improving long-term outcomes in this vulnerable population.

Zaleha Md Isa is a public health and epidemiologist at the Faculty of Medicine, Universiti Kebangsaan Malaysia (UKM).

Suhaini Kadiman, MD, MMed (Anaest) is a senior consultant anaesthesiologist at the National Heart Institute, Kuala Lumpur, Malaysia, with extensive experience in cardiac anaesthesia and perioperative imaging and management of complex cardiac surgical cases.

## Supplementary Information

Supplementary Material 1

## Data Availability

No datasets were generated or analysed during the current study.

## Abbreviations

AV: Atrioventricular
BAV: Bicuspid Aortic Valve
CAH: Cryopreserved Aortic Homograft
CI: Confidence Interval
CoNS: Coagulase—negative staphylococci
CPB: Cardiopulmonary Bypass
CT: Computed Tomography
EF: Ejection Fraction
ESC: European Society of Cardiology
HR: Hazard Ratio
IE: Infective Endocarditis
IFB: Intervalvular Fibrous Body
IVDU: Intravenous Drug User
MDT: Multidisciplinary Team
MVP: Mitral Valve Prolapse
NBTE: Non—bacterial Thrombotic Endocarditis
OR: Odds Ratio
PET: Positron Emission Tomography
PVE: Prosthetic Valve Endocarditis
RR: Risk Ratio
SPECT: Single—photon Emission Computed Tomography
TAVI: Transcatheter Aortic Valve Implantation
TEE: Transoesophageal Echocardiography
TIA: Transient Ischaemic Attack
TTE: Transthoracic Echocardiography
