# Supplementary material for: Valve-specific structural vulnerability in infective endocarditis: a narrative review of surgical complexity and outcomes
Source: J Cardiothorac Surg. 2026 Jul 17;21:800. doi: 10.1186/s13019-026-04523-w (PMC13673629; doi:10.1186/s13019-026-04523-w)
Supplement: Supplementary file 1 [file 13019_2026_4523_MOESM1_ESM.docx]

| **Valve Type** | **Anatomical Risk Factors** | **Haemodynamic Mechanism** | **Infection Pattern** | **Extension Risk** | **Vulnerability Grade** |
| --- | --- | --- | --- | --- | --- |
| **Aortic (Native)** | Fibrous skeleton continuity; age-related calcification; bicuspid aortic valve (1–2% prevalence) | Sustained high shear stress (systolic pressure ~120 mmHg); turbulent flow across calcified leaflets | Leaflet vegetation; peri-annular abscess (30–40%); aorto-cavitary fistula (1.6–3.2%) | High: intervalvular fibrous body, mitral valve, conduction system, ventricular septum | **Very High** |
| **Mitral (Native)** | Aortomitral continuity; mitral valve prolapse (2–3% prevalence); rheumatic valve disease; regurgitant jet lesions | Conditional vulnerability requiring pre-existing structural abnormality; regurgitant jet impact on atrial leaflet surface | Leaflet perforation; chordal rupture; annular abscess (10–15%); extension from aortic IE | Moderate: aortomitral continuity and left atrial wall | **Moderate–High** |
| **Tricuspid (Native)** | Relative anatomical isolation from fibrous skeleton; simpler chordal architecture | Low-pressure environment (right atrial pressure 0–8 mmHg); minimal shear stress | Predominantly associated with intravenous drug use; repeated bacteraemia; *Staphylococcus aureus* (60–80%); septic pulmonary emboli | Low: extension beyond the valve apparatus is uncommon | **Low** *(High in IVDU-associated IE)* |
| **Pulmonary (Native)** | Lowest-pressure valve in the cardiac cycle; minimal calcification; simple semilunar structure | Pulmonary artery pressure ~25/8–12 mmHg; minimal endothelial injury | Rare; associated with congenital heart disease, intravenous drug use, or pulmonary artery catheters; <2% of all IE cases | Very low: usually confined to the valve | **Very Low** |
| **Prosthetic (Any Position)** | Sewing-ring biofilm formation; peri-prosthetic dehiscence; avascular prosthetic material | Biofilm-mediated infection with impaired antimicrobial penetration; early (nosocomial) versus late (community-acquired) PVE | Sewing-ring infection; peri-annular abscess (40–60%); dehiscence causing severe regurgitation | Very high: extensive peri-annular destruction, fistula formation, annular disruption | **Highest** |

***Abbreviations:*** *IFB, intervalvular fibrous body; IE, infective endocarditis; IVDU, intravenous drug user; PVE, prosthetic valve endocarditis.*

**Table 1. Comparative Structural Vulnerability Across Cardiac Valves in Infective Endocarditis.**Vulnerability grade represents a conceptual synthesis of anatomical susceptibility, haemodynamic stress, propensity for peri-annular extension, and anticipated surgical complexity. It is intended as a qualitative framework rather than a validated prognostic score.

| **Valve Type** | **Extent of Structural Destruction** | **Preferred Surgical Technique** | **Alternative Surgical Technique** | **Special Considerations** |
| --- | --- | --- | --- | --- |
| **Aortic (Localised Disease)** | Leaflet vegetation without peri-annular extension | Aortic valve replacement (bioprosthetic or mechanical) | Leaflet repair with pericardial patch (rarely feasible) | Prosthesis selection remains debated; some centres favour bioprosthetic valves during active infection |
| **Aortic (Peri-annular Disease)** | Abscess confined to the annulus without intervalvular fibrous body involvement | Aortic valve replacement with patch reconstruction of the abscess cavity (pericardial or homograft patch) | Homograft root replacement | Patch reconstruction may be feasible when annular integrity is preserved and the abscess cavity is limited |
| **Aortic (Extensive Disease / IFB Involvement)** | Intervalvular fibrous body destruction, aorto-cavitary fistula, or extensive peri-annular abscess | Homograft root replacement, Bentall procedure, or Commando procedure | Prosthetic composite graft with patch reconstruction | Homografts may offer resistance to reinfection; Commando procedure is reserved for extensive intervalvular fibrous body involvement |
| **Mitral (Leaflet-Limited Disease)** | Leaflet perforation, chordal rupture, without annular involvement | Mitral valve repair (pericardial patch reconstruction, chordal replacement, annuloplasty) | Mitral valve replacement (usually bioprosthetic) | Repair is preferred whenever feasible and is associated with lower early mortality compared with replacement |
| **Mitral (Annular Disease)** | Annular abscess or extensive leaflet destruction | Mitral valve replacement with annular debridement and reconstruction | Mitral valve repair with patch reconstruction in selected cases | Bioprosthetic valves are frequently preferred in active infection |
| **Tricuspid (Vegetation-Predominant Disease)** | Vegetation without extensive leaflet destruction | Initial medical therapy; surgery when indicated using repair or annuloplasty | Tricuspid valve replacement (bioprosthetic) | Surgery is generally reserved for refractory infection, severe tricuspid regurgitation with right ventricular failure, or recurrent septic pulmonary emboli |
| **Tricuspid (IVDU-Associated Extensive Disease)** | Extensive destruction in active intravenous drug users with ongoing high reinfection risk | Tricuspid valvectomy as a bridge strategy | Valve repair when feasible and abstinence is achievable | Delayed valve replacement may be considered after infection eradication and sustained abstinence |
| **Prosthetic Valve Endocarditis (Limited Disease)** | Sewing-ring infection without extensive peri-annular destruction | Redo valve replacement with patch reconstruction of limited defects | Homograft root replacement | Complete removal of prosthetic material is mandatory |
| **Prosthetic Valve Endocarditis (Extensive Disease)** | Peri-annular abscess, prosthetic dehiscence, or fistula formation | Cryopreserved aortic homograft root replacement | Prosthetic composite graft with extensive patch reconstruction | Homograft root replacement is often preferred for extensive destruction; TAVI explantation remains technically demanding |

***Abbreviations****: AVR, aortic valve replacement; CAH, cryopreserved aortic homograft; IFB, intervalvular fibrous body; IVDU, intravenous drug user; PVE, prosthetic valve endocarditis; TAVI, transcatheter aortic valve implantation.*

**Table 2. Surgical Technique Selection According to Extent of Structural Destruction in Infective Endocarditis**. Surgical technique selection is influenced by the extent of structural destruction, peri-annular involvement, patient-specific factors, causative organism, and institutional expertise. Recommendations represent commonly reported approaches within contemporary surgical series and should not be interpreted as prescriptive treatment algorithms.

| **Valve/Procedure** | **Cardiopulmonary Bypass Time (min)** | **Cross-Clamp Time (min)** | **30-Day Mortality (%)** | **5-Year Survival (%)** | **10-Year Freedom from Reoperation (%)** | **Key References** |
| --- | --- | --- | --- | --- | --- | --- |
| **Aortic Homograft Root Replacement** | 214 ± 80 | 164 ± 56 | 18–22 | 58–65 | 85–90 | [22,80] |
| **Aortic Commando Procedure / IFB Reconstruction** | 236.9 ± 11.1 | Not reported | 28.3 *(90-day mortality: 37.0%)* | Not reported | Not reported | [78] |
| **Mitral Valve Repair** | 90–120 | 60–90 | 3.7 *(pooled estimate)* | 75–80 | 80–85 | [8,86,87] |
| **Mitral Valve Replacement** | 100–130 | 70–100 | 10.9 *(pooled estimate)* | 60–70 | 90–95 | [8,90] |
| **Tricuspid Valve Repair** | 60–90 | 40–60 | 12.5 | 63.9 | >90 *(non-IVDU populations)* | [90] |
| **Tricuspid Valvectomy** | 40–60 | 30–45 | 13 | Not reported *(bridge procedure)* | Not applicable | [40,45] |
| **Prosthetic Valve Endocarditis Surgery (Any Valve Position)** | 166 *(mean)* | Not reported | 17–26 | 56 | ~91 | [10,11,61,95] |
| **Redo Aortic Valve Replacement (Recurrent PVE or Structural Valve Deterioration)** | Not reported | Not reported | 15–21 | Not reported | Not applicable | [95] |

***Abbreviations:*** *IFB, intervalvular fibrous body; IVDU, intravenous drug user; PVE, prosthetic valve endocarditis.*

**Table 3. Perioperative Outcomes According to Valve Type and Surgical Approach in Infective Endocarditis.** Values are reported as mean ± standard deviation, mean values, pooled estimates, or ranges derived from the cited studies. Mortality estimates are based predominantly on observational cohort studies and meta-analyses. Freedom-from-reoperation estimates for tricuspid valve surgery exclude patients with ongoing intravenous drug use, in whom reinfection rates may reach 20–40% within 5 years. Outcomes should be interpreted in the context of substantial heterogeneity in patient characteristics, operative eras, and surgical techniques across studies.
